# Supplementary material for: Modeling glioblastoma heterogeneity as a dynamic network of cell states
Source: Mol Syst Biol. 2021 Sep 16;17(9):e10105. doi: 10.15252/msb.202010105 (PMC8444284; doi:10.15252/msb.202010105)
Supplement: Supplementary file 5 — Source Data for Figure 3 [file MSB-17-e10105-s001.zip › Figure3A_sourcedata/GSEA_3065/hallmarks_state4.GseaPreranked.1623416296068/HALLMARK_MYOGENESIS.html]

Details for gene set HALLMARK\_MYOGENESIS[GSEA]

|  || Dataset | state4 |
| Phenotype | NoPhenotypeAvailable |
| Upregulated in class | na\_pos |
| GeneSet | HALLMARK\_MYOGENESIS |
| Enrichment Score (ES) | 0.30821553 |
| Normalized Enrichment Score (NES) | 1.2383871 |
| Nominal p-value | 0.09328358 |
| FDR q-value | 0.22709839 |
| FWER p-Value | 0.828 |
Table: GSEA Results Summary

  

Fig 1: Enrichment plot: HALLMARK\_MYOGENESIS      
 Profile of the Running ES Score & Positions of GeneSet Members on the Rank Ordered List

  

| PROBE | GENE SYMBOL | GENE\_TITLE | RANK IN GENE LIST | RANK METRIC SCORE | RUNNING ES | CORE ENRICHMENT || 1 | CRYAB |  |  | 10 | 0.494 | 0.0626 | Yes |
| 2 | GABARAPL2 |  |  | 50 | 0.297 | 0.0950 | Yes |
| 3 | FHL1 |  |  | 62 | 0.286 | 0.1304 | Yes |
| 4 | CLU |  |  | 95 | 0.252 | 0.1580 | Yes |
| 5 | CNN3 |  |  | 160 | 0.217 | 0.1758 | Yes |
| 6 | MYL6B |  |  | 210 | 0.201 | 0.1941 | Yes |
| 7 | CDKN1A |  |  | 319 | 0.171 | 0.1989 | Yes |
| 8 | WWTR1 |  |  | 370 | 0.159 | 0.2115 | Yes |
| 9 | SORBS1 |  |  | 406 | 0.151 | 0.2255 | Yes |
| 10 | BHLHE40 |  |  | 451 | 0.143 | 0.2370 | Yes |
| 11 | PDE4DIP |  |  | 457 | 0.143 | 0.2548 | Yes |
| 12 | SPARC |  |  | 539 | 0.132 | 0.2589 | Yes |
| 13 | ITGB4 |  |  | 680 | 0.117 | 0.2515 | Yes |
| 14 | SIRT2 |  |  | 709 | 0.113 | 0.2616 | Yes |
| 15 | PFKM |  |  | 825 | 0.102 | 0.2563 | Yes |
| 16 | CHRNB1 |  |  | 856 | 0.099 | 0.2644 | Yes |
| 17 | OCEL1 |  |  | 907 | 0.095 | 0.2686 | Yes |
| 18 | SLC6A8 |  |  | 926 | 0.093 | 0.2779 | Yes |
| 19 | FKBP1B |  |  | 945 | 0.092 | 0.2869 | Yes |
| 20 | SSPN |  |  | 1013 | 0.087 | 0.2875 | Yes |
| 21 | RIT1 |  |  | 1021 | 0.086 | 0.2976 | Yes |
| 22 | DTNA |  |  | 1063 | 0.084 | 0.3019 | Yes |
| 23 | MEF2C |  |  | 1107 | 0.082 | 0.3055 | Yes |
| 24 | ITGB5 |  |  | 1183 | 0.077 | 0.3035 | Yes |
| 25 | MRAS |  |  | 1215 | 0.075 | 0.3082 | Yes |
| 26 | EIF4A2 |  |  | 1311 | 0.070 | 0.3020 | No |
| 27 | SPTAN1 |  |  | 1371 | 0.067 | 0.3013 | No |
| 28 | MAPRE3 |  |  | 1625 | 0.058 | 0.2679 | No |
| 29 | DMD |  |  | 1710 | 0.055 | 0.2615 | No |
| 30 | EFS |  |  | 1730 | 0.054 | 0.2655 | No |
| 31 | CKB |  |  | 1774 | 0.053 | 0.2655 | No |
| 32 | GSN |  |  | 1935 | 0.049 | 0.2459 | No |
| 33 | CRAT |  |  | 2077 | 0.045 | 0.2290 | No |
| 34 | SORBS3 |  |  | 2193 | 0.042 | 0.2158 | No |
| 35 | FLII |  |  | 2293 | 0.039 | 0.2049 | No |
| 36 | PDLIM7 |  |  | 2389 | 0.037 | 0.1944 | No |
| 37 | AGL |  |  | 2452 | 0.036 | 0.1891 | No |
| 38 | ABLIM1 |  |  | 2473 | 0.035 | 0.1905 | No |
| 39 | DMPK |  |  | 2474 | 0.035 | 0.1950 | No |
| 40 | FGF2 |  |  | 3038 | 0.024 | 0.1073 | No |
| 41 | TGFB1 |  |  | 3283 | 0.019 | 0.0703 | No |
| 42 | EPHB3 |  |  | 3397 | 0.016 | 0.0540 | No |
| 43 | HDAC5 |  |  | 3461 | 0.013 | 0.0456 | No |
| 44 | AGRN |  |  | 3832 | -0.001 | -0.0141 | No |
| 45 | ADCY9 |  |  | 4112 | -0.016 | -0.0570 | No |
| 46 | GAA |  |  | 4119 | -0.017 | -0.0558 | No |
| 47 | ATP6AP1 |  |  | 4313 | -0.030 | -0.0831 | No |
| 48 | NOTCH1 |  |  | 4321 | -0.031 | -0.0802 | No |
| 49 | AKT2 |  |  | 4464 | -0.041 | -0.0978 | No |
| 50 | MYLK |  |  | 4481 | -0.043 | -0.0948 | No |
| 51 | TSC2 |  |  | 4530 | -0.046 | -0.0965 | No |
| 52 | ACSL1 |  |  | 4583 | -0.050 | -0.0984 | No |
| 53 | IFRD1 |  |  | 4671 | -0.057 | -0.1051 | No |
| 54 | SCD |  |  | 4717 | -0.061 | -0.1044 | No |
| 55 | ERBB3 |  |  | 4720 | -0.061 | -0.0967 | No |
| 56 | AEBP1 |  |  | 4733 | -0.062 | -0.0905 | No |
| 57 | PRNP |  |  | 4778 | -0.066 | -0.0890 | No |
| 58 | ADAM12 |  |  | 4896 | -0.075 | -0.0982 | No |
| 59 | PLXNB2 |  |  | 4962 | -0.080 | -0.0983 | No |
| 60 | SOD3 |  |  | 5006 | -0.083 | -0.0945 | No |
| 61 | STC2 |  |  | 5150 | -0.097 | -0.1050 | No |
| 62 | COL4A2 |  |  | 5156 | -0.098 | -0.0931 | No |
| 63 | APP |  |  | 5192 | -0.101 | -0.0856 | No |
| 64 | TAGLN |  |  | 5209 | -0.102 | -0.0750 | No |
| 65 | NCAM1 |  |  | 5391 | -0.117 | -0.0889 | No |
| 66 | TPM3 |  |  | 5409 | -0.120 | -0.0761 | No |
| 67 | NQO1 |  |  | 5566 | -0.138 | -0.0834 | No |
| 68 | NAV2 |  |  | 5634 | -0.150 | -0.0747 | No |
| 69 | ITGA7 |  |  | 5715 | -0.163 | -0.0665 | No |
| 70 | FDPS |  |  | 5828 | -0.190 | -0.0599 | No |
| 71 | IGFBP7 |  |  | 5868 | -0.199 | -0.0404 | No |
| 72 | MYH9 |  |  | 5874 | -0.200 | -0.0152 | No |
| 73 | ITGB1 |  |  | 5877 | -0.201 | 0.0106 | No |
| 74 | IGFBP3 |  |  | 6184 | -0.397 | 0.0128 | No |
Table: GSEA details [plain text format]

  

Fig 2: HALLMARK\_MYOGENESIS: Random ES distribution      
 Gene set null distribution of ES for **HALLMARK\_MYOGENESIS**
